# Symmetry‐Driven Unconventional Magnetoelectric Coupling in Perovskite Altermagnets: From Bulk to the Two‐Dimensional Limit

**DOI:** 10.1002/advs.202600004

**Published:** 2026-04-15

**Authors:** Zhou Cui, Ziye Zhu, Xunkai Duan, Bowen Hao, Xianzhang Chen, Jiayong Zhang, Tong Zhou

**Affiliations:** ^1^ Ningbo Institute of Digital Twin Eastern Institute of Technology Ningbo Zhejiang China; ^2^ School of Physics and Astronomy Shanghai Jiao Tong University Shanghai China; ^3^ School of Physical Science and Technology Suzhou University of Science and Technology Suzhou China

**Keywords:** altermagnets, magnetoelectric coupling, multiferroics, perovskites, two‐dimensional materials

## Abstract

The emergence of altermagnets establishes a new paradigm for multiferroics. Unlike conventional multiferroics that rely on direct magnetoelectric coupling, multiferroic altermagnets host a crystal‐symmetry‐mediated magnetoelectric interaction that is intrinsically more efficient and robust. Among candidate material platforms, layered perovskites are particularly appealing owing to their structural diversity and synthetic versatility. However, magnetoelectric properties at the two‐dimensional scale remain largely unexplored, hindering their applicability in miniaturized, highly integrated devices. Here, we systematically investigate the dimensional evolution of ferroelectric polarization and magnetism in perovskite systems through symmetry analysis. We demonstrate that altermagnetism can persist in the two‐dimensional limit, yet is strongly constrained by the magnetic configuration—with only C‐type antiferromagnetic order supporting it. On the basis of mode‐decomposition calculations, we further reveal that symmetry‐restricted multimode couplings simultaneously govern ferroelectric polarization and altermagnetic spin splitting. Finally, combined with first‐principles calculations, we propose several strategies to lift the magnetic‐configuration constraint, extending the range of viable altermagnetic systems. These results underscore the critical role of dimensionality in symmetry‐driven magnetoelectric coupling in perovskite altermagnets and pave the way toward next‐generation electrically controlled spintronic and multiferroic devices.

## Graphical abstract

The symmetry‐driven coexistence of altermagnetism and (anti)ferroelectricity in perovskites shows a strong dimensional dependence. Upon reducing the system from bulk to the two‐dimensional limit, only C‐type antiferromagnetic order retains ferroelectrically switchable altermagnetism, whereas A‐ and G‐type orders become conventional antiferromagnets. This dimensional evolution offers a clear pathway for efficient electrical control of magnetism in low‐dimensional spintronic and multiferroic devices.

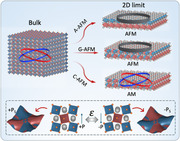

## 1 Introduction

Magnetoelectric multiferroics, in which spin polarization can be manipulated by an electric field and vice versa, are highly sought after for applications in high‐density, low‐energy devices [1, 2, 3]. However, this prospect has been limited by the intrinsically weak magnetoelectric coupling in conventional multiferroics, arising from the fundamental dichotomy of P‐ and T‐symmetry breaking in electric and magnetic orders [4, 5]. Recently, unconventional magnets, particularly altermagnets (AM) that unify features of ferromagnets and antiferromagnets, have attracted widespread attention [6, 7, 8, 9, 10, 11, 12, 13, 14, 15, 16, 17, 18, 19, 20, 21, 22, 23, 24, 25, 26, 27, 28, 29, 30, 31, 32, 33, 34, 35, 36, 37, 38], while also providing a new research paradigm for multiferroics [39, 40, 41, 42, 43, 44, 45, 46, 47, 48, 49, 50, 51, 52, 53, 54, 55, 56, 57]. Altermagnetism is characterized by spin‐group symmetry and is governed by real‐space crystal symmetry [58, 59, 60, 61], a fundamental constraint that is also shared by ferroelectricity. This symmetry compatibility enables a crystal‐symmetry‐mediated unconventional magnetoelectric coupling, which is inherently more efficient and robust than conventional mechanisms.

Perovskites constitute a large family of compounds with versatile physical properties and are among the most extensively studied materials in condensed matter physics and materials science [62]. In the context of multiferroics, perovskites are widely regarded as some of the most promising candidates for practical applications, exemplified by ${\mathrm{BiFeO}}_{3}$ and its related compounds [63]. Notably, perovskites also provide a key platform for multiferroic altermagnets, including antiferroelectric altermagnets (AFEAM) [39], e.g., ${\mathrm{GdFeO}}_{3}$‐type ${\mathrm{BiCrO}}_{3}$ [39], and ferroelectric altermagnets (FEAM) [42], e.g., the Ruddlesden–Popper (RP) phase ${\mathrm{Ca}}_{3}$ ${\mathrm{Mn}}_{2}$ $O_{7}$ [40, 41]. Meanwhile, functional electronic devices are predominantly realized in thin‐film or low‐dimensional geometries, making the investigation of material properties in the two‐dimensional (2D) limit crucial for device miniaturization and high‐density integration in the post‐Moore era [64, 65]. However, existing studies of perovskite multiferroic altermagnets have thus far focused almost exclusively on bulk crystalline phases. This raises the following questions: Can these perovskite materials retain multiferroic altermagnetic order in the 2D limit, and how does crystal symmetry evolve across the dimensional crossover?

In this work, we perform a systematic symmetry analysis of RP phase and ${\mathrm{GdFeO}}_{3}$‐type perovskites to provide an in‐depth investigation of dimensionality effects on the ferroelectric and altermagnetic properties at both bulk and 2D scales. We find that these two bulk structures can be converted into the same 2D FEAM perovskites via exfoliation and surface effects; however, the emergence of altermagnetism is constrained by the magnetic configuration, with only the C‐type antiferromagnetic order preserved. The ferroelectric polarization originates from the bulk specific alternating in‐plane polarization, while the $m_{z}$ symmetry plays a crucial role in enabling altermagnetism. Furthermore, mode‐decomposition calculations for the Ca–Mn–O system reveal that symmetry‐restricted multimode couplings simultaneously govern ferroelectric polarization and altermagnetic spin splitting in 2D perovskite multiferroic altermagnets.

More intriguingly, we discover that the crystal symmetry can be further engineered to lift the magnetic‐configuration constraint in the 2D limit through several strategies, including superlattice engineering, shear strain, electric fields, and substrate engineering. Combined with first‐principles calculations, these approaches can convert other magnetic configurations into altermagnets and even realize ferroelectric fully compensated ferrimagnets, thereby extending the range of viable unconventional multiferroics. These results clarify the underlying mechanism of unconventional magnetoelectric coupling in perovskite multiferroic altermagnets and establish a pathway for designing electrically controllable 2D spintronic and multifunctional devices [66, 67, 68, 69, 70, 71, 72, 73, 74, 75].

## 2 Results and Discussions

**FIGURE 1:**
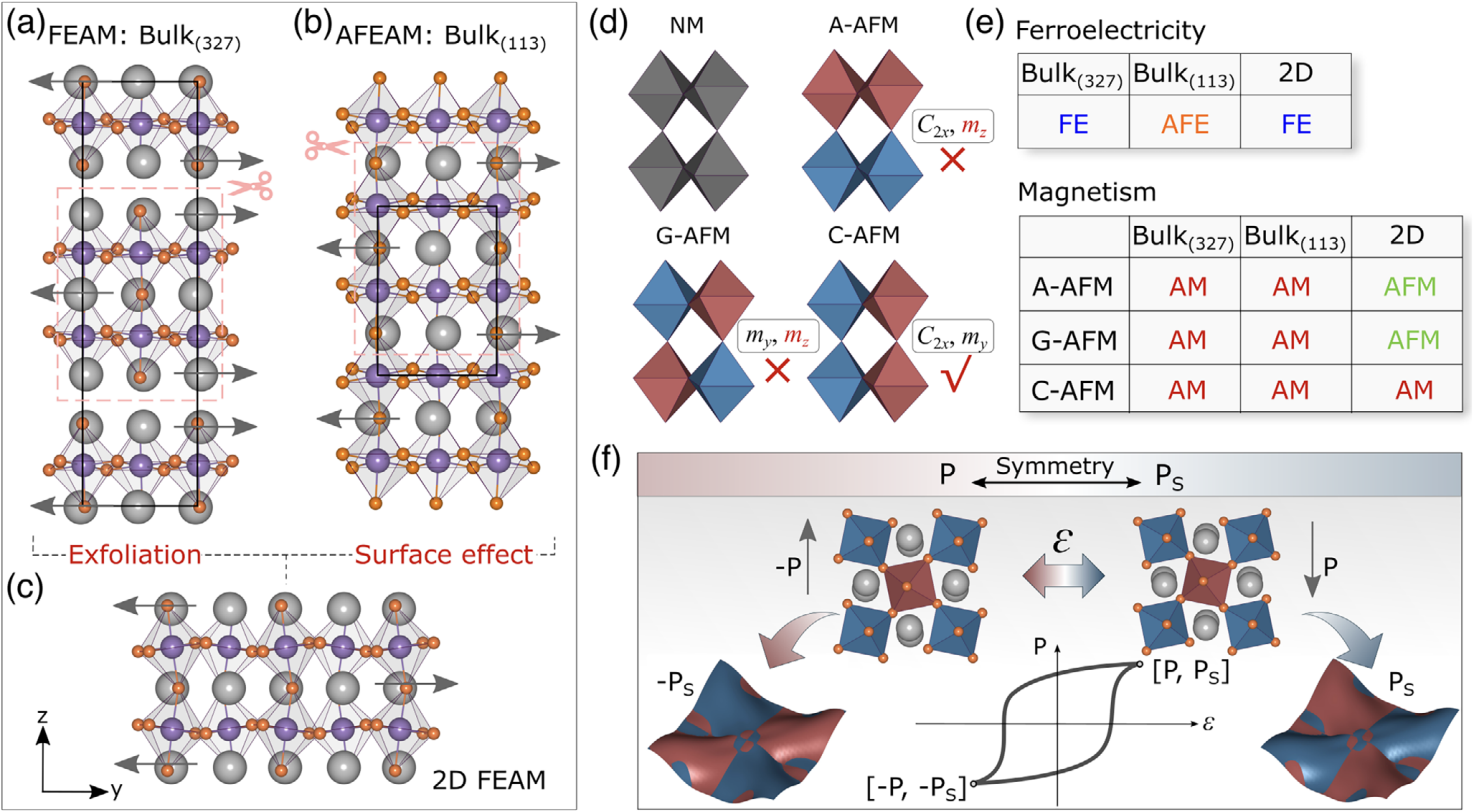
(a) Crystal structures of bulk FEAM Ruddlesden–Popper perovskites (space group: ${\mathrm{Cmc}2}_{1}$, labeled as ${\mathrm{Bulk}}_{\left(327\right)}$) and (b) AFEAM ${\mathrm{GdFeO}}_{3}$‐type perovskites (space group: Pnma, labeled as ${\mathrm{Bulk}}_{\left(113\right)}$). Black arrows indicate the direction of ferroelectric polarization, and black solid lines delineate the crystallographic unit cell. (c) Two‐dimensional slabs derived from the bulk structures via exfoliation or surface‐induced dimensional reduction. (d) Schematic of real‐space symmetry operations connecting opposite‐spin sublattices in magnetic octahedra under different magnetic configurations in 2D perovskites, red and blue denote opposite spin channels. (e) Summary of ferroelectric and magnetic orders in bulk and 2D perovskites. (f) Schematic illustration of symmetry‐driven unconventional magnetoelectric coupling in 2D perovskite altermagnets.

Bulk RP phase perovskites with the chemical formula $A_{3}$ $B_{2}$ $O_{7}$ (hereafter denoted as ${\mathrm{Bulk}}_{\left(327\right)}$) crystallize into a van der Waals (vdW) layered structure with the polar space group *Cmc2* 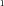, as shown in Figure 1a. Relative to the high‐symmetry parent I4/mmm structure, the RP phase is strongly distorted, featuring A‐site cation displacements, ${\mathrm{BO}}_{6}$ octahedral rotations and tilts, as well as Jahn–Teller distortions. Among these, the A‐site displacements primarily generate ferroelectric polarization (see Figure S1), while the B‐site cations carry the magnetic moments. This symmetry reduction simultaneously satisfies the criteria for altermagnetism, rendering ${\mathrm{Bulk}}_{\left(327\right)}$ a switchable FEAM [40]. In contrast, bulk ${\mathrm{GdFeO}}_{3}$‐type perovskites with the general formula ${\mathrm{ABO}}_{3}$ (denoted as ${\mathrm{Bulk}}_{\left(113\right)}$) adopt the *Pnma* space group. Although ${\mathrm{Bulk}}_{\left(113\right)}$ exhibits lattice distortions similar to those in ${\mathrm{Bulk}}_{\left(327\right)}$, the antiparallel displacements of adjacent A‐site layers cancel out, resulting in an AFEAM state [39], as illustrated in Figure 1b.

Advanced fabrication techniques, such as molecular beam epitaxy and oxide membrane exfoliation, have enabled the growth of freestanding perovskite oxides down to the monolayer limit [76, 77, 78], paving the way for the design of functional properties like ferroelectricity [79]. Remarkably, both exfoliation from ${\mathrm{Bulk}}_{\left(327\right)}$ [80, 81] and surface engineering of ${\mathrm{Bulk}}_{\left(113\right)}$ [82] yield the same 2D perovskite structure with the chemical formula $A_{6}$ $B_{4}$ $O_{14}$ (hereafter simply denoted as 2D), as shown in Figure 1c. To avoid surface or interface reconstructions associated with the so‐called “polar catastrophe” [83, 84], we focus on A‐site cations with +2 charge, ensuring that each AO layer remains charge neutral. In this well‐defined 2D structure, the absence of cancellation between adjacent A‐site layers gives rise to a net in‐plane ferroelectric polarization, as illustrated in Figure 1c,e. Indeed, the ferroelectricity in ${\mathrm{Bulk}}_{\left(113\right)}$ exhibits a pronounced even‐odd layer dependence, as discussed in Figure S2.

The magnetic properties exhibit a qualitatively different dimensional dependence. In bulk, both ${\mathrm{Bulk}}_{\left(327\right)}$ and ${\mathrm{Bulk}}_{\left(113\right)}$ display altermagnetism under A‐, G‐, and C‐AFM orderings, while in the 2D limit only the C‐AFM state preserves altermagnetism, as illustrated in Figure 1e. Accordingly, in 2D perovskites, the coexistence of ferroelectricity and altermagnetism is restricted to the magnetic configuration.

From a symmetry perspective, altermagnetism requires that the two opposite‐spin sublattices are connected by crystallographic rotation (R) or mirror (m) transformations, possibly combined with (rather than directly connected by) translation (t) or inversion (I) symmetries [12]. In 2D systems, the additional $m_{z}$ and $C_{2z}$ symmetries are also excluded [85]. Bulk perovskites possess eight unitary symmetry operations in their geometric space groups: $\left\{E,C_{2y},m_{x},m_{z}\right\}$ for ${\mathrm{Bulk}}_{\left(327\right)}$, each followed by two appropriate lattice translations; and $\left\{E,I,C_{2x},C_{2y},C_{2z},m_{x},m_{y},m_{z}\right\}$ for ${\mathrm{Bulk}}_{\left(113\right)}$. When considering different AFM configurations, half of these operations are excluded, as listed in Table 1. Importantly, the remaining operations do not involve direct t or I symmetries that would forbid altermagnetism, ensuring that all A‐, G‐, and C‐AFM exhibit altermagnetic behavior. For the 2D structure, the geometric space group contains four symmetry operations, $\left\{E,C_{2y}t,m_{x}t,m_{z}\right\}$, which are also reduced by half when magnetic order is taken into account. For A‐ and G‐type AFM configurations, the remaining operations include the $m_{z}$ symmetry, enforcing a conventional AFM state. While for the C‐AFM configuration, the inherent $m_{z}$ symmetry is preserved, preventing direct connection between opposite‐spin sublattices and thus satisfying the symmetry requirements for altermagnetism, as illustrated in Figure 1d and Table 1.

**TABLE 1:** Symmetry operations excluded upon considering magnetic configurations in bulk and 2D perovskites (connecting opposite‐spin sublattices). Note that in ${\mathrm{Bulk}}_{\left(327\right)}$, the $m_{x}$ and $C_{2y}$ operations can each follow two distinct translation components.

Magnetic order	${\mathrm{Bulk}}_{\left(327\right)}$	${\mathrm{Bulk}}_{\left(113\right)}$	2D
A‐AFM	$\left\{C_{2y}t,\;m_{z},\;m_{z}t\right\}$	$\left\{C_{2y}t,\;C_{2z}t,\;m_{y}t,\;m_{z}t\right\}$	$\left\{C_{2x}t,\;m_{z}t\right\}$
C‐AFM	$\left\{C_{2y}t,\;m_{x}t\right\}$	$\left\{C_{2x}t,\;C_{2y}t,\;m_{x}t,\;m_{y}t\right\}$	$\left\{C_{2x}t,\;m_{y}t\right\}$
G‐AFM	$\left\{m_{x}t,\;m_{z},\;m_{z}t\right\}$	$\left\{C_{2x}t,\;C_{2z}t,\;m_{x}t,\;m_{z}t\right\}$	$\left\{m_{y}t,\;m_{z}t\right\}$

**FIGURE 2:**
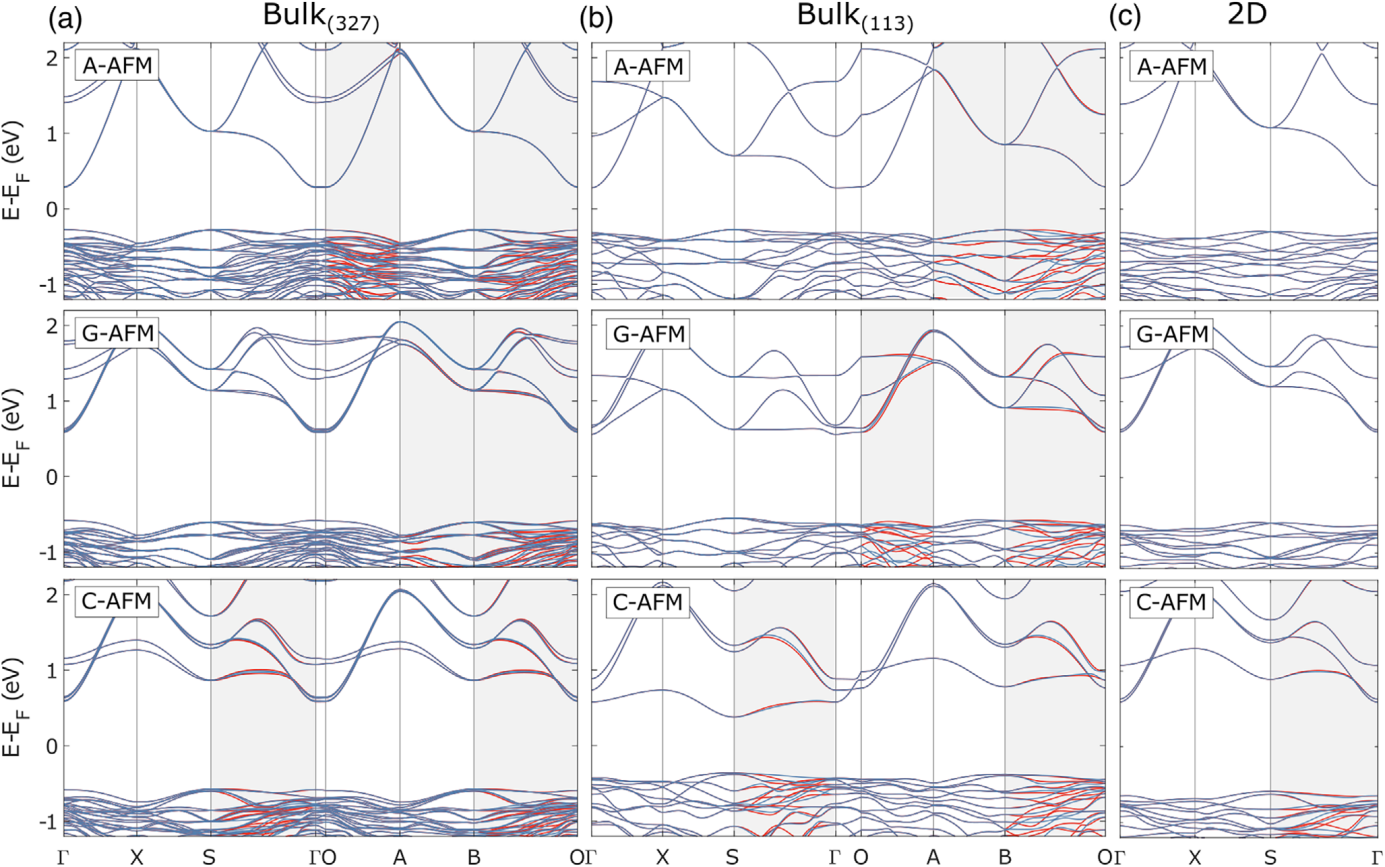
Spin‐resolved band structures of the Ca‐Mn‐O multiferroic altermagnets for (a) ${\mathrm{Bulk}}_{\left(327\right)}$, (b) ${\mathrm{Bulk}}_{\left(113\right)}$, and (c) 2D structure. A‐, G‐, and C‐type AFM orders are considered. Red (blue) lines correspond to spin‐up (spin‐down) channels. Gray shaded regions highlight momentum paths exhibiting spin splitting. The corresponding first Brillouin zones are provided in Figure S3.

Next, focusing on the Ca–Mn–O system, we combine symmetry analysis with first‐principles electronic‐structure calculations to elucidate the dimensional dependence of unconventional magnetoelectric coexistence and coupling in perovskite multiferroic altermagnets. All compounds in this system are experimentally realized, including the ${\mathrm{Bulk}}_{\left(327\right)}$ (${\mathrm{Ca}}_{3}$ ${\mathrm{Mn}}_{2}$ $O_{7}$) and ${\mathrm{Bulk}}_{\left(113\right)}$ (${\mathrm{CaMnO}}_{3}$) phases [86, 87]. Figure 2 shows the spin‐resolved band structures of the three different structures under various AFM configurations. Consistent with the previous analysis, the bulk systems all exhibit momentum‐dependent spin splitting, whereas in the 2D limit only the C‐type AFM, which is also the magnetic ground state (as shown in Table S1), retains altermagnetism. Furthermore, the paths of band splitting vary with the magnetic configuration, as dictated by symmetry. The spin‐dependent bands can be described by the Kohn–Sham equation

(1) $$\left[\frac{1}{2}{\left(k-i\nabla \right)}^{2}+V_{\sigma }\right]\psi_{\sigma }\left(k\right)=E_{\sigma }\left(k\right)\psi_{\sigma }\left(k\right)$$

where k is the wave vector and $V_{\sigma }$ the effective potential for spin σ=↑,↓. An exchange operation O is defined as a symmetry element of the geometric space group that maps spin‐up to spin‐down, $OV_{↑}O^{-1}=V_{↓}$. If Ok=k, the spin is degenerate at k; if instead $Ok=k^{\prime }$, we have $E_{↑}\left(k\right)\neq E_{↓}\left(k\right)=E_{↓}\left(k^{\prime }\right)$, indicating spin splitting with opposite spins at k and $k^{\prime }$, characteristic of altermagnetism [88, 89].

As an example, for bulk ${\mathrm{CaMnO}}_{3}$ with C‐AFM order, the symmetry operations $O\in \{C_{2x}t,C_{2y}t,m_{x}t,m_{y}t\}$, possibly in combination with I, enforce spin degeneracy along the high‐symmetry paths Γ—X—S and Γ—O—A—B, whereas along S—Γ and B—O, no O maps k onto itself, giving rise to altermagnetic spin splitting. The specific high‐symmetry points are labeled in Figure S3. For A‐ and G‐type AFM, different sets of O alter the spin‐splitting paths accordingly. Similarly, for a 2D slab with C‐AFM order, $O\in \{C_{2y}t,m_{x}t\}$ maps the opposite‐spin sublattices, producing spin splitting along Γ—S, while bands remain spin‐degenerate along Γ—X—S. In contrast, for A‐ and G‐AFM, all k points are mapped onto themselves by O, resulting in conventional AFM.

**FIGURE 3:**
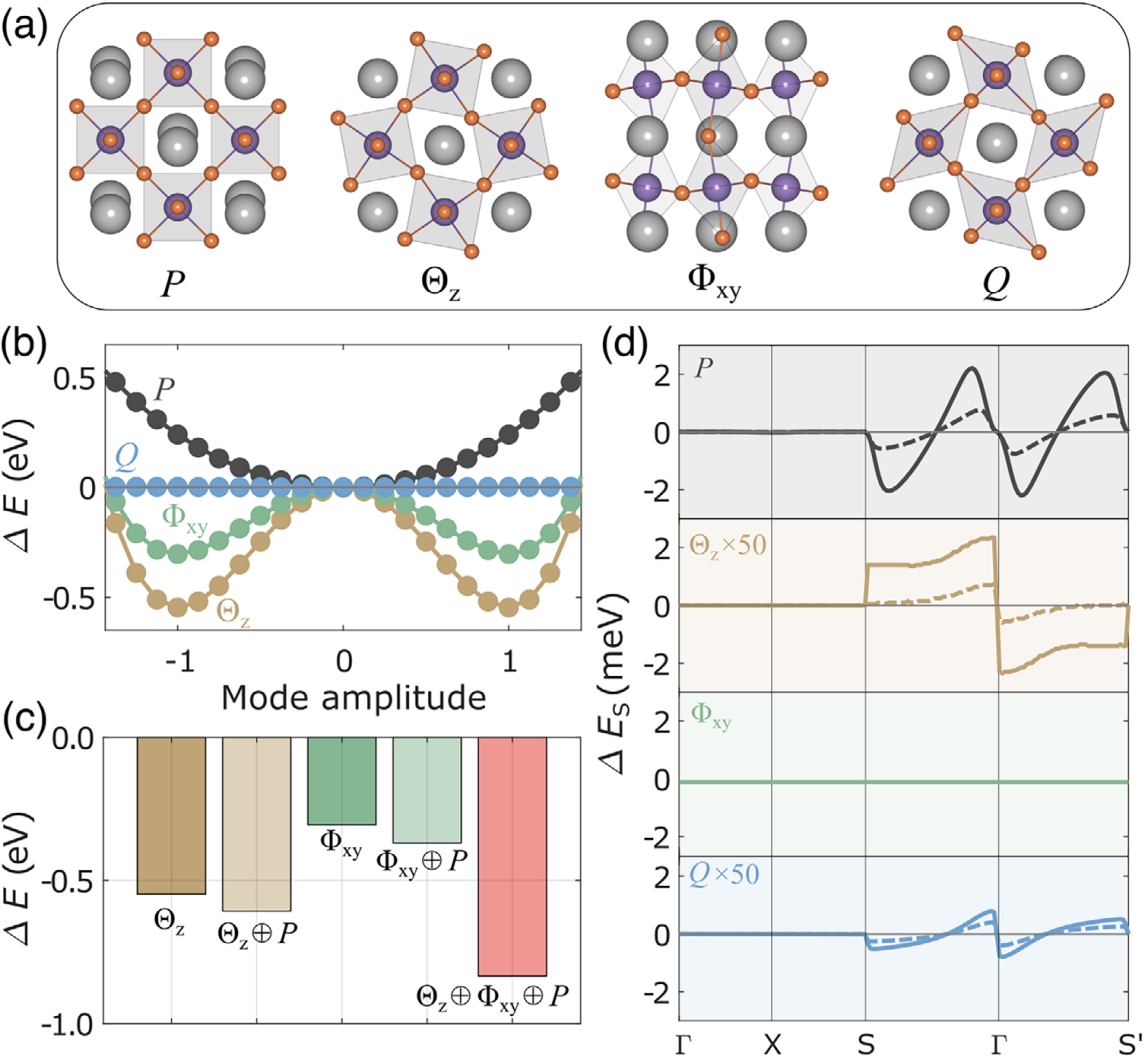
(a) Major structural distortions of the ground state in the studied 2D perovskites, including in‐plane ferroelectric polarization P, in‐plane octahedral rotation $\Theta_{z}$, octahedral tilting $\Phi_{xy}$, and Jahn–Teller distortion Q. (b) Energy surface as a function of the amplitude of each mode for the 2D high symmetry parent P4/mmm phase in the Ca‐Mn‐O system. (c) Energy gain associated with different lattice distortion modes acting on the P4/mmm phase. (d) Altermagnetic spin splitting as a function of the amplitude of different distortion modes; dashed and solid lines correspond to mode amplitudes of 0.5 and 1 (DFT‐relaxed structures), respectively.

To gain more insight into the microscopic origin of the magnetoelectric effect in 2D perovskite multiferroic altermagnets, we analyze the different lattice distortion modes. Through mode decomposition, we identify four major atomic distortions: in‐plane polarization distortion P, in‐phase oxygen octahedral rotations $\Theta_{z}$, out‐of‐plane oxygen octahedral tilts $\Phi_{xy}$, and the Jahn‐Teller lattice distortion Q, as illustrated in Figure 3a and Figure S4. We project the contribution of each mode onto the ground state structure and calculate the energy surface around the high‐symmetry parent P4/mmm reference structure in the Ca‐Mn‐O system. Figure 3b shows the total energy as a function of the amplitude for each individual distortion. Relatively large energy gains can be seen within characteristic double‐well potentials for the $\Theta_{z}$ and $\Phi_{xy}$ distortions, whereas the in‐plane polarization P remains stable. Figure 3c further demonstrates that the collective $\Theta_{z}⊕\Phi_{xy}⊕P$ combination strongly lowers the total energy, indicating that ferroelectricity in this system arises from a multimode coupling effect, analogous to hybrid improper ferroelectricity [90, 91].

We then evaluate the contribution of each distortion mode to the spin splitting, as shown in Figure 3d. The results show that the P, $\Theta_{z}$, and Q modes all contribute positively to the altermagnetic spin splitting, such that larger distortion amplitudes lead to larger spin splittings, whereas the $\Phi_{xy}$ mode does not induce spin splitting due to symmetry constraints. Notably, the spin splitting generated by the three modes individually, when summed (∼2 meV), remains much smaller than that of the ground state (∼20 meV), indicating that altermagnetic spin splitting in the 2D Ca–Mn–O structure originates from the cooperative contribution of multiple distortion modes. In addition, spin‐resolved band structures for different B‐site elements are calculated, and the distortion amplitudes of the various modes are quantitatively analyzed (see Figure S5). These results further demonstrate a strong correlation between the magnitude of altermagnetic spin splitting and the multimode coupling effect.

At this stage, we have clarified the dimensional dependence and microscopic origin of the magnetoelectric coupling in perovskite multiferroic altermagnets, with the key finding that altermagnetism can persist in the 2D limit but is strongly constrained by the magnetic configuration, such that only the C‐AFM order preserves it. Through A‐ and B‐site elemental substitution calculations, we further find that many 2D perovskite materials do not stabilize the C‐AFM order, as shown in Figure S6. This raises the question: can this magnetic‐configuration constraint be lifted?

**FIGURE 4:**
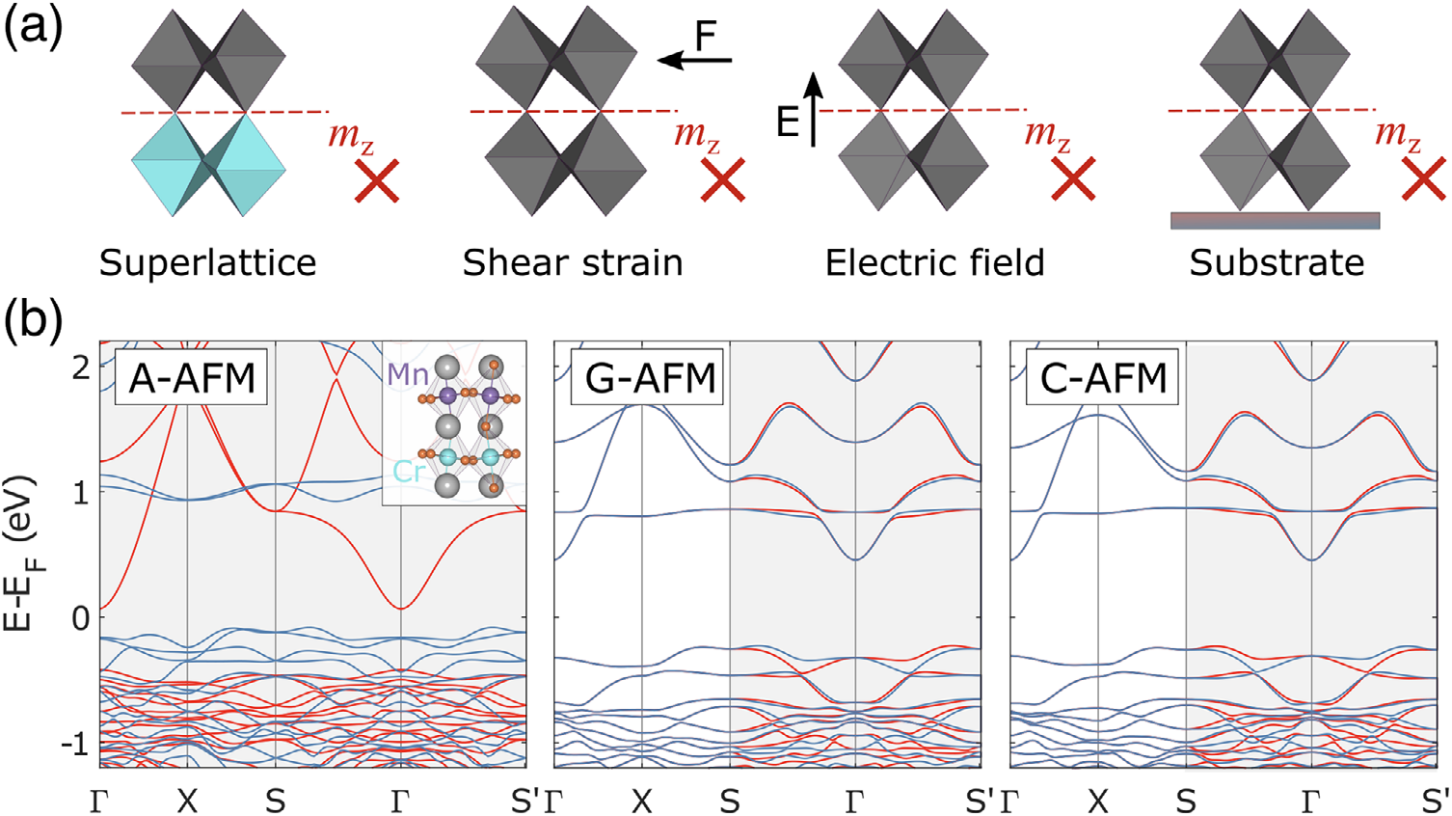
(a) Several strategies to break $m_{z}$ symmetry in real space, including superlattice engineering, shear strain, electric field, and substrate engineering. (b) Spin‐resolved band structures of different magnetic orders in the 2D Ca‐Mn/Cr‐O superlattice (chemical formula ${\mathrm{Ca}}_{6}$ ${\mathrm{Mn}}_{2}$ ${\mathrm{Cr}}_{2}$ $O_{14}$).

In general, there are two rules to design altermagnetism: (i) breaking specific symmetries without considering the magnetic order; and (ii) preserving specific symmetries after the magnetic configuration is established, thereby preventing them from serving to connect opposite‐spin sublattices. These specific symmetries include t and I (and $m_{z}$, $C_{2z}$ in the 2D case). In both cases, the R‐related rotational symmetries must still be preserved. As discussed in Figure 1d and Table 1, the emergence of C‐AFM as the 2D altermagnetic state arises precisely from the second mechanism: the inherent $m_{z}$ symmetry remains intact, preventing it from serving as the symmetry operation that connects opposite‐spin sublattices. Once the magnetic configuration is identified as a non–C‐AFM state, the second mechanism is no longer applicable, and $m_{z}$ symmetry must be broken in the crystal space to realize altermagnetism. Here, we propose four general strategies to achieve this, including superlattice engineering, shear strain, electric fields, and substrate engineering, as illustrated in Figure 4a. In Figure 4b and Figure S7, we show the band structures obtained after applying the aforementioned strategies. We find that, upon breaking the $m_{z}$ symmetry, G‐AFM order in 2D perovskites transforms from a conventional AFM into AM. Interestingly, under the reduced symmetry, A‐AFM order exhibits the behavior of a fully compensated ferrimagnet–zero net magnetization but spin splitting across the entire Brillouin zone, as the opposite‐spin sublattices are no longer connected by any symmetry operation. To our knowledge, this represents the first report of a 2D ferroelectric fully compensated ferrimagnet.

## 3 Conclusion

In summary, based on symmetry analysis and first‐principles calculations, we have systematically explored the dimensional evolution and microscopic origin of unconventional magnetoelectric coupling in perovskite multiferroic altermagnets. We further propose a series of potential strategies to lift the magnetic‐configuration constraint and even realize 2D ferroelectric fully compensated ferrimagnets. Notably, in the 2D perovskite multiferroic altermagnets considered here, an applied electric field can reverse the spin splitting, as illustrated in Figure 1f. Such robust, electric‐field–controllable magnetism and spin polarization in the 2D limit can be readily integrated into van der Waals heterostructures or exploited through the altermagnetic proximity effect, thereby providing a versatile platform for emerging magnetic‐field‐free quantum phenomena [66, 67, 68, 69, 70, 71, 72, 73, 74, 75]. Moreover, by leveraging the intrinsic advantages of altermagnets, multiferroic altermagnets can substantially outperform conventional multiferroics in terms of spin dynamics, read/write speed, and high‐density integration, offering promising opportunities for next‐generation memory technologies.

## 4 Experimental Section

For our calculations, we performed density functional theory (DFT) as implemented in the Vienna Ab‐initio Simulation Package (VASP) code [92]. The interactions between valence electrons and ionic cores were described using the projector augmented‐wave (PAW) pseudopotentials. The Perdew–Burke–Ernzerhof (PBE) exchange‐correlation functional was used for structural relaxation. The on‐site Coulomb and exchange parameters for Mn ions were set to U = 4.5 eV and J = 1.0 eV, respectively [90]. Electron wavefunctions were expanded in a plane‐wave basis set with a kinetic energy cutoff of 600 eV, and the convergence criterion for energy was set to 10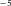 eV. The Brillouin zones of bulk and 2D slab were sampled using 7 × 7 × 3 and 7 × 7 × 1 Monkhorst‐Pack meshes, respectively. Geometry relaxations of the structural models were carried out until the residual force on each atom was less than 5 × 10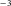 $\mathrm{eV}{Å}^{-1}$. For the 2D slab calculations, a sufficiently large vacuum thickness of 20 Å was set in order to avoid the interlayer interactions between neighboring periodic images. The FE polarization can be calculated using: 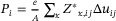. Here, $P_{i}$ is the polarization component along the i‐direction with units of C/m, and A is the unit cell area of the 2D system. The summation is over the atomic index κ, with 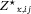 being the Born effective charge tensor component for atom κ. The Born effective charge tensor is calculated through density functional perturbation theory (DFPT). $\Delta u_{j}$ is the displacement of atom κ along the j‐direction.

## Author Contributions

Z. Cui and Z. Y. Zhu contributed equally to this work. Z. Cui and Z. Y. Zhu were involved in writing the original draft, data curation, software, and formal analysis. X. K. Duan and B. W. Hao contributed to software, data curation, and validation. X. Z. Chen and J. Y. Zhang participated in formal analysis, and reviewing and editing. Z. Y. Zhu and T. Zhou were responsible for conceptualization, supervision, project administration, funding acquisition, and reviewing and editing.

## Conflicts of Interest

The authors declare no conflicts of interest.

## Supporting information

Supporting file

## Data Availability

The data that support the findings of this study are available from the corresponding author upon reasonable request.
