# Supplementary material for: Symmetry‐Driven Unconventional Magnetoelectric Coupling in Perovskite Altermagnets: From Bulk to the Two‐Dimensional Limit
Source: Adv Sci (Weinh). 2026 Apr 15:e00004. Online ahead of print. doi: 10.1002/advs.202600004 (PMC13335491; doi:10.1002/advs.202600004)
Supplement: Supplementary file 1 — Supporting file [file ADVS-9999-e00004-s001.pdf]

Supporting Information for

Symmetry-Driven Unconventional Magnetoelectric Coupling in Perovskite Altermagnets: From Bulk to the Two-Dimensional Limit

Zhou Cui[1], *Ziye Zhu*[1,*], *Xunkai Duan*[1,2], *Bowen Hao*[1], *Xianzhang Chen*[1], *Jiayong Zhang*[1,3], *Tong Zhou*[1,*]

Email Address: zyzhu@eitech.edu.cn (Dr. Ziye Zhu); tzhou@eitech.edu.cn (Prof. Tong Zhou)

[1]Ningbo Institute of Digital Twin, Eastern Institute of Technology, Ningbo, Zhejiang 315200, China
[2]School of Physics and Astronomy, Shanghai Jiao Tong University, Shanghai 200240, China
[3]School of Physical Science and Technology, Suzhou University of Science and Technology, Suzhou, 215009, China

Table S1: The relative energies(meV/u.c.) of bulk and 2D Ca-Mn-O perovskite systems with different magnetic configurations, all referenced to the respective ground states.

	AAFM	CAFM	GAFM	FM
$\text{Bulk}_{(113)}$	23.31	74.97	73.48	**0**
$\text{Bulk}_{(327)}$	12.19	1.74	**0**	32.91
2D	4.77	**0**	6.52	16.30

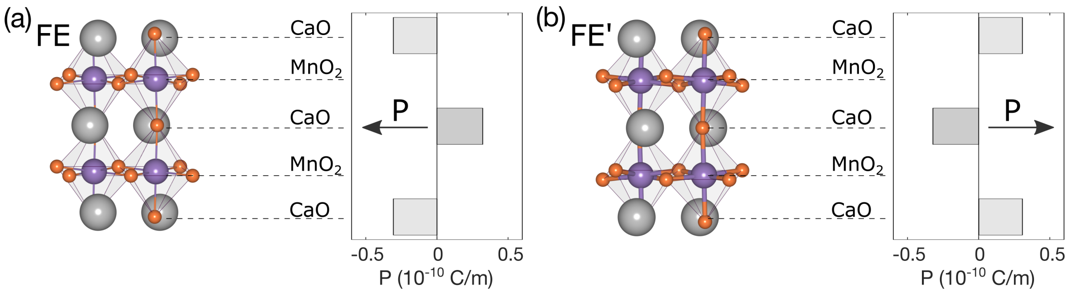

Figure S1: The layer-by-layer projection of the polarization for 2D $Ca_6Mn_4O_{14}$ with two ferroelectric configurations with opposite polarization directions FE and FE′ states. The ferroelectric polarization arises predominantly from ionic displacements in the CaO layers (approximately 0.31×10^{-10} C/m), with only minor contributions from the MnO_6 octahedra.

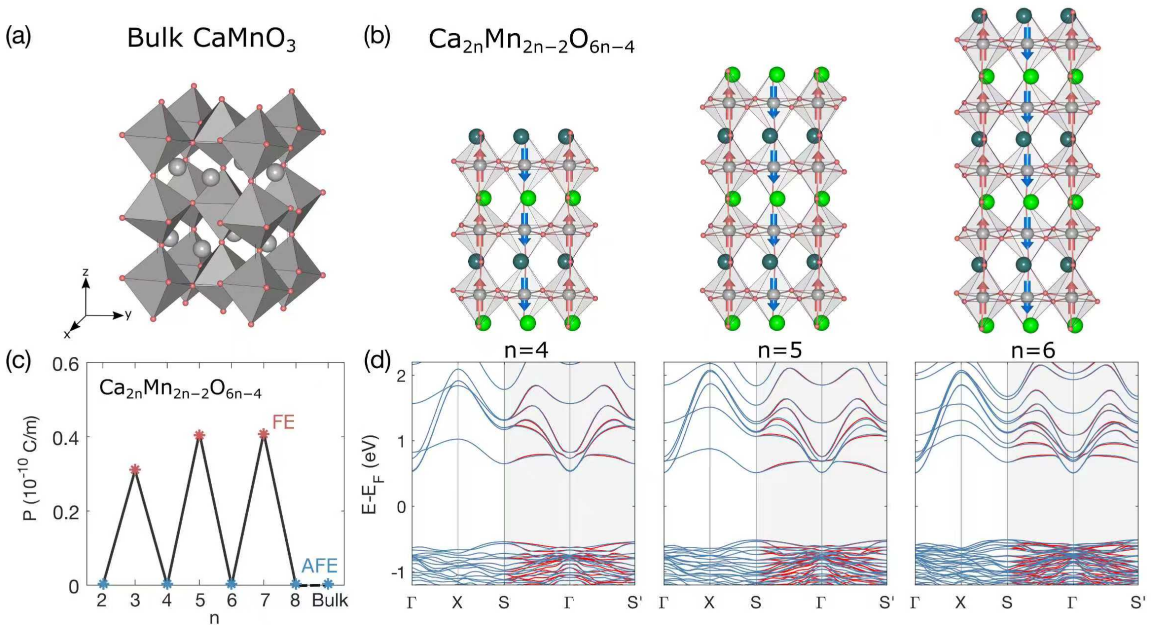

Figure S2: Layer-dependent FE polarization and AM spin splitting of $CaMnO_3$ thin films. (a) Crystal structure of bulk $CaMnO_3$. (b) Schematic of the 2D $Ca_{2n}Mn_{2n-2}O_{6n-4}$ (n = 2, 4, 6) obtained by cleaving bulk $CaMnO_3$. (c) Layer-dependent FE polarization of $CaMnO_3$ thin films with different layers. (d) Layer-dependent AM spin splitting of $CaMnO_3$ thin films with different layers. The odd-number-layer systems exhibit a net ferroelectric polarization, whereas even-number-layer systems display antiferroelectric characteristics due to the mutual cancellation of antipolar displacements between adjacent CaO layers. In striking contrast to the strong layer-number dependence observed for ferroelectricity, the AM spin splitting remains robust and nearly unaffected by layer parity, demonstrating inherent stability against variations in film thickness.

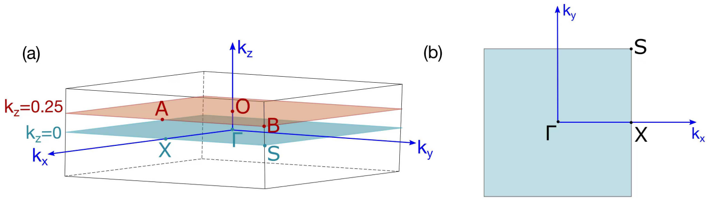

Figure S3: Brillouin zones of the Ca-Mn-O perovskite system for (a) bulk and (b) 2D slab.

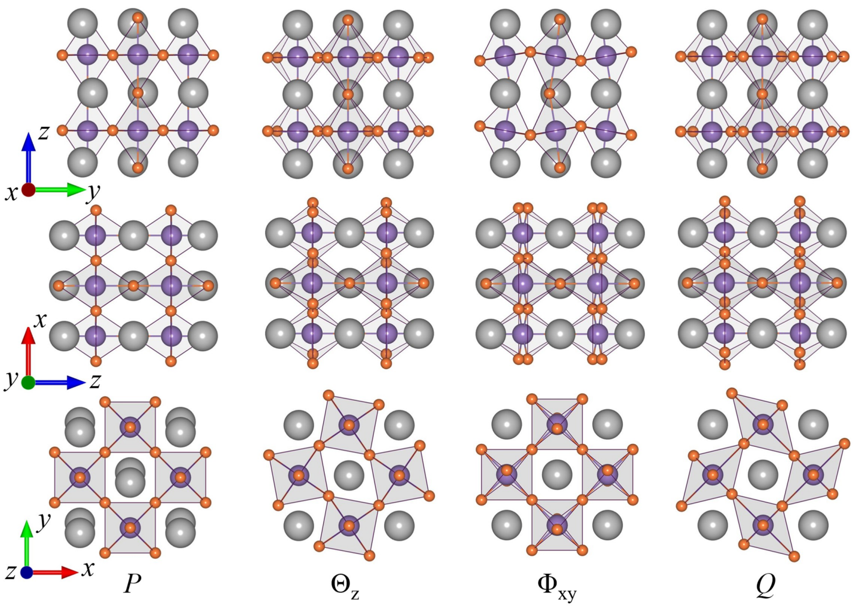

Figure S4: The 3D illustrations of the structural distortion modes.

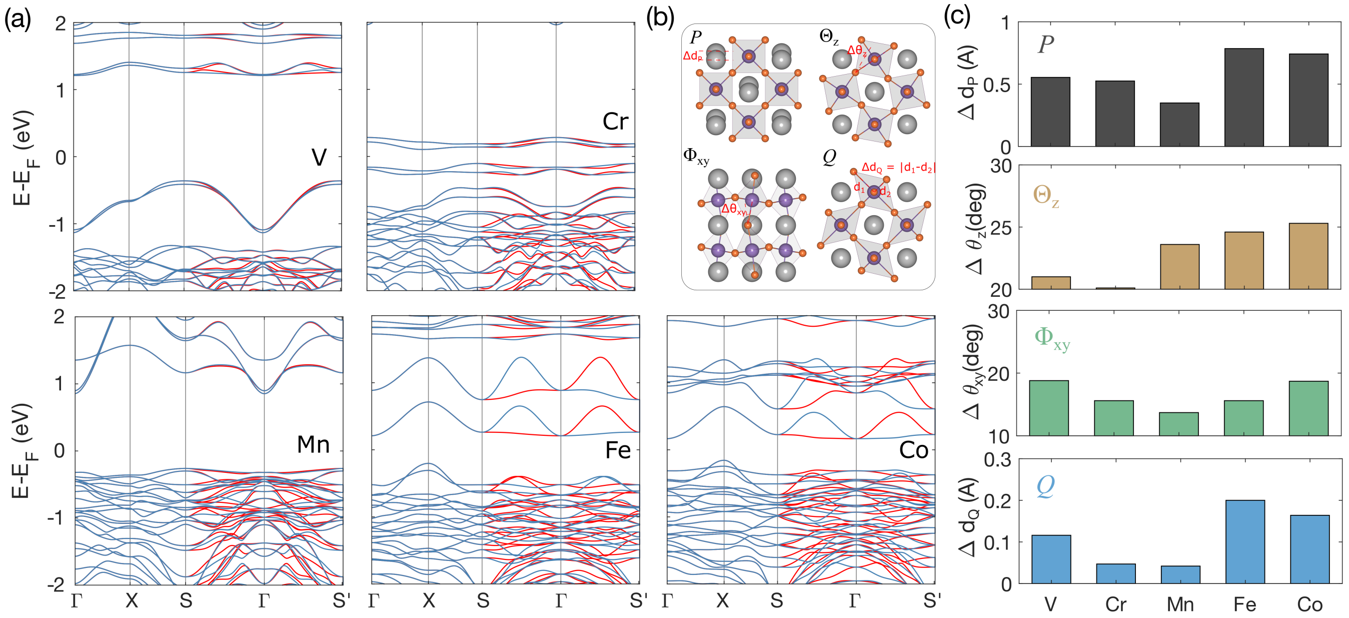

Figure S5: Effect of atomic distortions on the AM spin splitting of 2D Ca-X-O (X = V, Cr, Mn, Fe, Co) perovskite systems. (a) Band structures of 2D Ca-X-O systems. (b) Four distinct atomic distortions, including polarization distortion P about the in-plane; in-phase oxygen octahedral rotations Θ_z about the out-of-plane; antiphase oxygen octahedral rotations Φ_{xy}; Jahn-Teller lattice distortion Q. Δd_P represents the average displacement of Ca atoms; $\Delta\theta_z$ and $\Delta\theta_{xy}$ denote the in-plane and out-of-plane rotation angles of the oxygen octahedra, respectively; while Δd_Q quantifies the axial length difference of an octahedron. (c) The calculated Δd_P, $\Delta\theta_z$, $\Delta\theta_{xy}$ and Δd_Q of 2D Ca-X-O systems. The significant spin splittings observed in 2D Ca-Fe-O and Ca-Co-O systems are primarily attributed to the combined effects of large Δd_P, $\Delta\theta_z$, and Δd_Q. In contrast, although the 2D Ca-V-O system exhibits the largest $\Delta\theta_{xy}$ among these materials, its AM spin splitting remains minimal. This suggests that the AM spin splitting in these 2D perovskite systems is primarily driven by sizable Δd_P, $\Delta\theta_z$, and Δd_Q, rather than by a large $\Delta\theta_{xy}$.

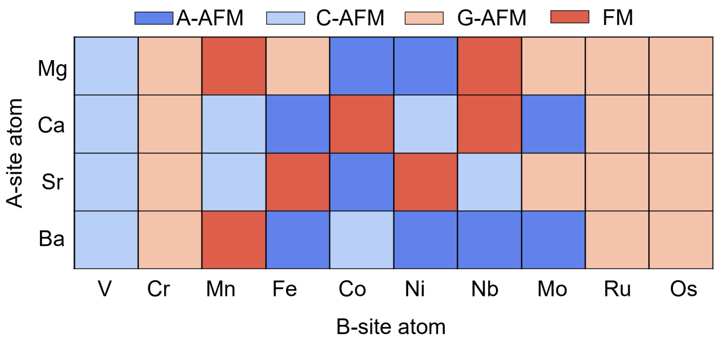

Figure S6: Magnetic ground-state configurations of 2D A-B-O perovskite systems, where A-site cations are Mg, Ca, Sr, or Ba atoms, and B-site cations include V, Cr, Mn, Fe, Co, Ni, Nb, Mo, Ru, and Os atoms, respectively.

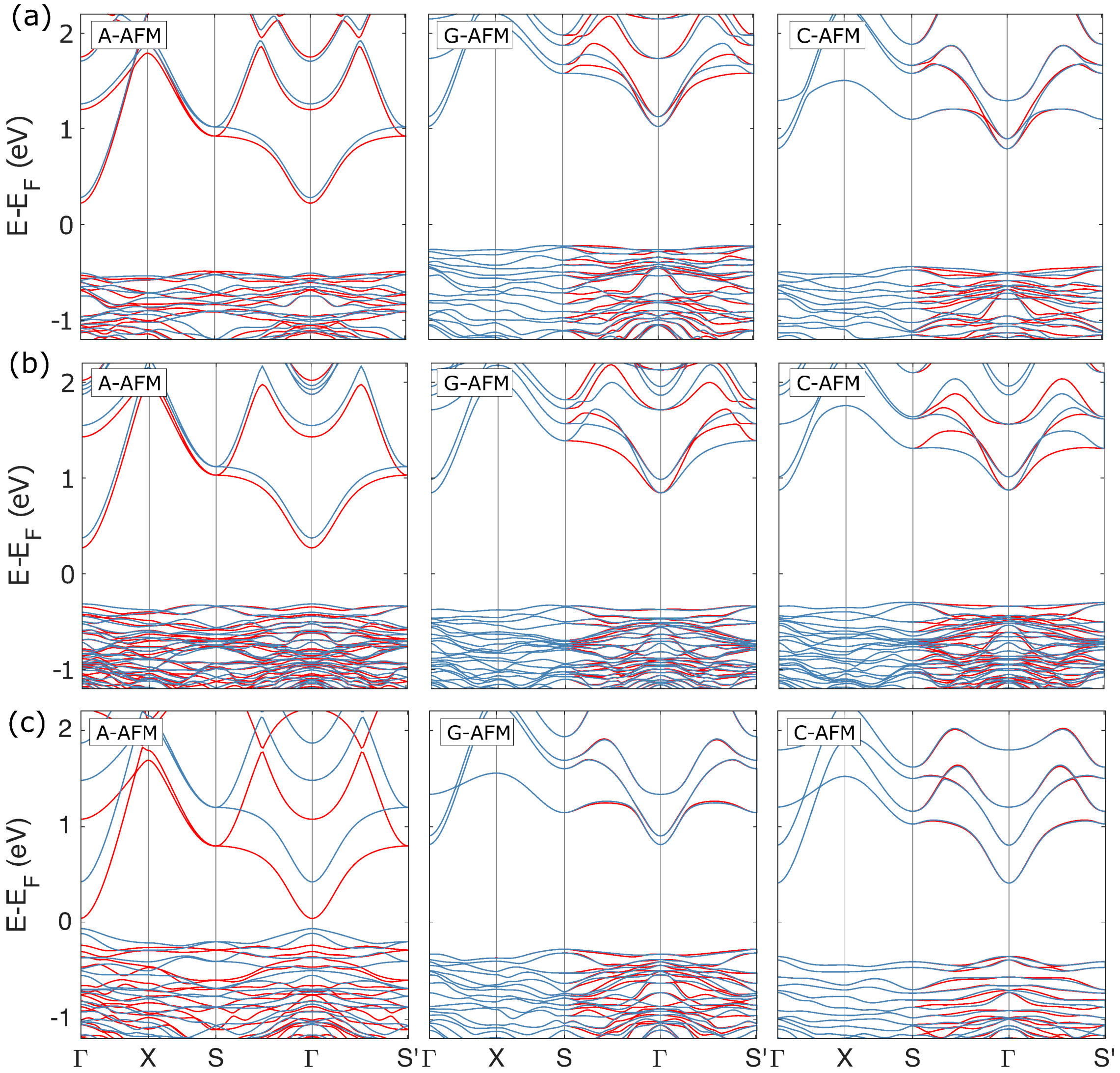

Figure S7: Spin-resolved band structures of different magnetic orders in the 2D Ca-Mn-O system with (a) shear strain, (b) non-magnetic substrate ($Ca_6Ti_4O_{14}$ here) and (c) electric-field of 0.1 eV/Å

.
